# Effects of the Ethanol Extract of *Dipterocarpus alatus* Leaf on the Unpredictable Chronic Mild Stress-Induced Depression in ICR Mice and Its Possible Mechanism of Action

**DOI:** 10.3390/molecules24183396

**Published:** 2019-09-18

**Authors:** Supawadee Daodee, Orawan Monthakantirat, Kanlaya Ruengwinitwong, Kankrittanon Gatenakorn, Juthamart Maneenet, Charinya Khamphukdee, Nazim Sekeroglu, Yaowared Chulikhit, Anake Kijjoa

**Affiliations:** 1 Division of Pharmaceutical Chemistry, Faculty of Pharmaceutical Sciences, Khon Kaen University, Khon Kaen 40002, Thailand; csupawad@kku.ac.th (S.D.); oramon@kku.ac.th (O.M.); k.ruengt@gmail.com (K.R.); kankrittanon@gmail.com (K.G.); juthamart_pp@hotmail.com (J.M.); charkh@kku.ac.th (C.K.); 2 Department of Horticulture, Faculty of Agriculture, Killis 7 Aralik University, Killis 79000, Turkey; nsekeroglu@gmail.com; 3 ICBAS-Instituto de Ciências Biomédicas Abel Salazar and CIIMAR, Rua de Jorge Viterbo Ferreira, 228, 4050-313 Porto, Portugal

**Keywords:** *Dipterocarpus alatus*, flavonoids, depression, UCMS, HPA axis, neurogenesis, MAO-A inhibition

## Abstract

Treatment of the unpredictable chronic mild stress (UCMS) mice with the ethanol extract of *Dipterocarpus alatus* leaf attenuated anhedonia (increased sucrose preference) and behavioral despair (decreased immobility time in tail suspension test (TST) and forced swimming test (FST)). The extract not only decreased the elevation of serum corticosterone level and the index of over-activation of the hypothalamic-pituitary-adrenal (HPA) axis, caused by UCMS, but also ameliorated UCMS-induced up-regulation of serum- and glucocorticoid-inducible kinase 1 (SGK1) mRNA expression and down-regulation of cyclic AMP-responsive element binding (CREB) and brain-derived neurotrophic factor (BDNF) mRNAs in frontal cortex and hippocampus. In vitro monoamine oxidase (MAO) inhibition assays showed that the extract exhibited the partial selective inhibition on MAO-A. HPLC analysis of the extract showed the presence of flavonoids (luteolin-7-*O*-glucoside, kaempferol-3-glucoside, rutin) and phenolic acids (gallic acid, ferulic acid, and caffeic acid) as major constituents.

## 1. Introduction

Depression is one of the most debilitating and life-threatening mental disorders worldwide. Clinically, it is characterized by a loss of interest, depressed mood, anhedonia (reduced ability to feel pleasure from natural rewards), guilty conscience, irritability, difficulty to concentrate, poor appetite, insomnia, and suicidal tendencies [1]. Although the mechanism of depression has not been fully understood, a number of reports indicated that the reduction of the hippocampal serotonin (5-HT) neurotransmission is involved in unpredictable chronic mild stress (UCMS)-induced depression-like behavior [2]. It is well recognized that the development of depression is closely related to stress, which is, in turn, associated with the dysregulation of the HPA [3]. Excessive and prolonged chronic stress can impair the HPA axis negative feedback system, thus causing the hyperactivity and hypersecretion of glucocorticoids (GCs) from the adrenal glands. GCs, which are the most relevant biological factors in major depression disorder, also regulate stress responses [4]. Solid evidences have shown that chronically high concentrations of GCs could impair hippocampal neurogenesis [5]. Decreasing levels of neurotrophins such as brain-derived neurotrophic factor (BDNF) and nerve growth factor, which are closely linked to neuronal cell survival and neurogenesis, could lead to depressive behavior [6]. Moreover, reduction of the BDNF expression also causes an atrophy and loss of hippocampal neurons [7]. Cellular stress response and neuronal function are also impacted by serum- and glucocorticoid-inducible kinase 1 (SGK1) [8]. SGK1 is also involved in depression by modulating the effects of GCs on neurogenesis and glucocorticoid receptor function [9]. On the other hand, the BDNF level and HPA axis function can be normalized by chronic treatment of antidepressants, partly by recovering the glucocorticoid receptor function, which can alleviate depressive symptoms [10,11].

*Dipterocarpus alatus* Roxb. ex G. Don, also known colloquially as the resin tree, is a tropical forest tree of a dense evergreen in tropical Asia. *D. alatus* is not only important for durable timber and useful oleo-resin (dammar) and varnishes but also known for its ethnomedicinal use. Bark of *D. alatus* is used to treat rheumatism and liver diseases, as well as to stimulate appetite in cattle [12]. In Ayurvedic medicine, oil from *D. alatus* is used for treatment of ulcerated wounds [13]. Oligostilbenoids from the stem wood of *D. alatus* were reported to exhibit inhibitory effects on acetylcholinesterase, a potential target for treating Alzheimer’s disease [14]. Recently, Yongram et al. reported the investigation of the antioxidant and cytotoxic activities of extracts of leaves, bark, twigs, and oleo-resin, as well as chemical composition of the oleo-resin from *D. alatus*, collected in Thailand [15]. On the other hand, chemical and pharmacological investigations of the leaf extract of *D. alatus* are scarce. In the investigation of the flavonoid patterns, by paper chromatography, of leaves of some species of *Dipterocarpus*, from Bangladesh, quercetin, apigenin, procyanidin, prodelphinidin, and quercetin-3-glucoside were found to be present in *D. alatus* leaf [16]. As flavonoids can exert antioxidant and anti-neuroinflammatory activities [17], stimulate pro-survival signaling cascades [18], restore the monoamine neurotransmitter, and promote hippocampal neurogenesis [19,20], whose mechanisms could be involved in the depression pathophysiology, the antidepressant effects of *D. alatus* leaf should be thoroughly investigated to warrant its capacity as a potential renewable resource for antidepressant agents.

Therefore, the effects of the ethanol extract of *D. alatus* leaf on depressive behaviors in the UCMS model of depression were investigated in this study. Additionally, the underlying molecular mechanism related to HPA axis function and neurogenesis-signaling pathway such as cyclic AMP-responsive element-binding (CREB), BDNF, and SGK1 mRNAs, was also explored. In order to substantiate the results obtained from the pharmacological studies, the HPLC fingerprint was carried out to identify the major constituents present in the *D. alatus* leaf extract.

## 2. Results

### 2.1. The Effect of the Ethanol Extract of D. Alatus Leaf (LE) on Anhedonia Behavior Using Sucrose Preference Test

**Figure 1:**
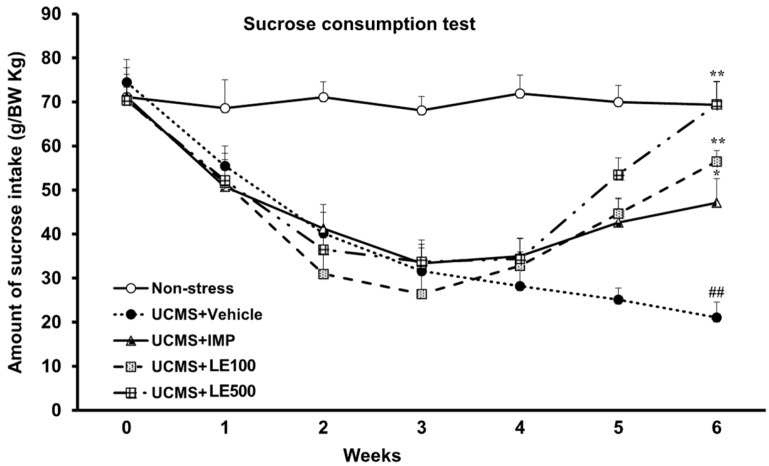
The effects of the ethanol extract of *D. alatus* leaf (LE) on the unpredictable chronic mild stress (UCMS)-induced anhedonia behavior in sucrose preference test. The amount of 2% sucrose solution taken by each animal group was measured as an index of anhedonia behavior once a week. Each consumption represents the mean ± S.E.M. (*n* = 10–12 in each animal group). ^##^ *p* < 0.001 vs. vehicle-treated non-stress group. ^*^ *p* < 0.05, ^**^ *p* < 0.001 vs. vehicle-treated UCMS group.

A sucrose preference test was aimed to examine the UCMS-induced anhedonia behavior. Sucrose consumption in the UCMS group was significantly reduced when compared with the non-stress group. Imipramine (IMP)-treated UCMS mice showed a significant increase in 2% sucrose intake at the 5th week. In the same way, the ethanol extract of *D. alatus* leaf (LE), at doses of 100 mg/kg and 500 mg/kg, showed a significant increase in the amount of 2% sucrose solution in the 5th and 6th week when compared with the vehicle-treated UCMS group (Figure 1). Treatment with the ethanol extract of *D. alatus* leaf (LE) caused the same attenuation of the anhedonia behavior in both UCMS mice and imipramine (IMP)-treated group (for detailed statistical analysis, see Supplementary materials, Table S1).

### 2.2. The Effect of the Ethanol Extract of D. Alatus Leaf (LE) on Hopeless Behavior Using Tail Suspension Test (TST) and Forced Swimming Test (FST)

**Figure 2:**
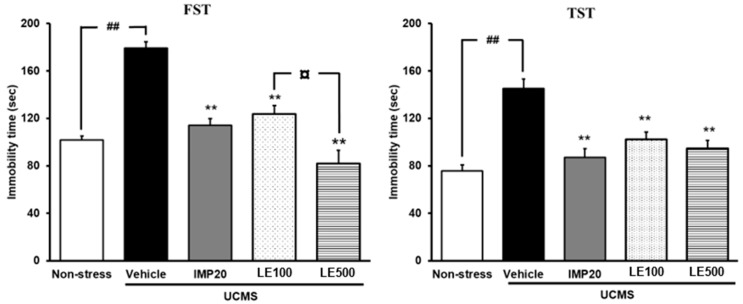
The effect of the ethanol extract of *D. alatus* leaf (LE) on hopeless behavior using forced swimming test (FST) and tail suspension test (TST). Immobility of each animal group was measured as an index of hopeless behavior 6 weeks after starting unpredictable chronic mild stress (UCMS) procedure. Each column represents the mean ± S.E.M. (*n* = 10–12 in each animal group). ^##^ *p* < 0.001 vs. the vehicle-treated non-stress group. ^**^ *p* < 0.001 vs. the vehicle-treated UCMS group. ^¤^ *p* < 0.05 compared between doses of the ethanol extract of *D. alatus* leaf (LE).

Hopeless behavior of UCMS mice was determined using tail suspension test (TST) and forced swimming test (FST). As shown in Figure 2, the vehicle-treated UCMS group displayed a significant increase in immobility time when compared with the vehicle-treated non-stress group. The ethanol extract of *D. alatus* leaf (LE) -treated UCMS group significantly decreased the immobility time when compared with the vehicle-treated UCMS group, similar to that treated by imipramine (IMP). Moreover, the ethanol extract of *D. alatus* leaf (LE)-treated UCMS group significantly decreased the immobility time in FST and TST, in dose-dependent manner (for detailed statistical analysis, see Supplementary materials, Tables S2 and S3).

**Figure 3:**
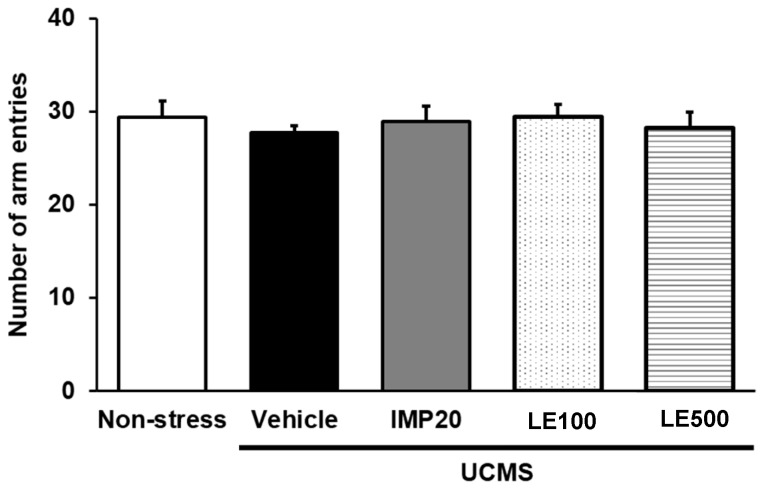
The effect of and the ethanol extract of *D. alatus* leaf (LE) on the locomotor activity in the Y-maze task. The number of arm entries of each group was determined. Each column represents the mean ± S.E.M. (*n* = 10–12 in each animal group).

A Y-maze task was also performed to evaluate the locomotor activity so that false positive results can be excluded. Results showed that neither imipramine (IMP) nor the ethanol extract of *D. alatus* leaf improved the locomotor activity of UCMS mice (Figure 3).

### 2.3. The Effect of the Ethanol Extract of D. Alatus Leaf (LE) on Serum Corticosterone (CORT) Levels in Unpredictable Chronic Mild Stress (UCMS) Mice

**Figure 4:**
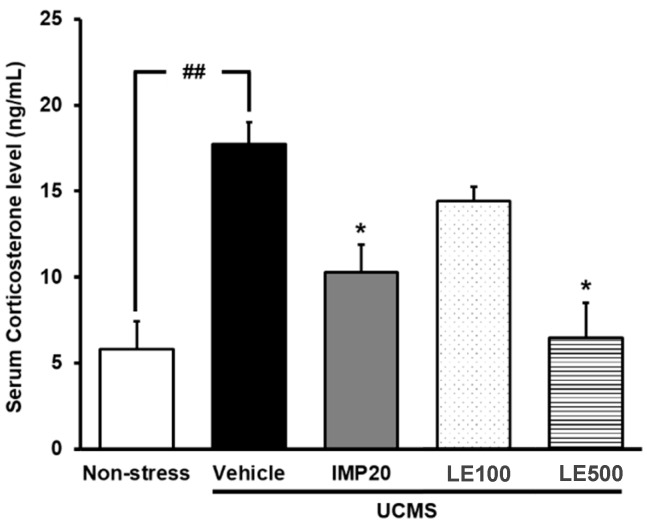
Effects of and the ethanol extract of *D. alatus* leaf (LE) and imipramine (IMP) on unpredictable chronic mild stress (UCMS)-induced elevation in serum corticosterone (CORT) levels. Each column represents the mean ± S.E.M. (*n* = 3–5). ^##^ *p*<0.001 vs. the sham-operated group. ^*^ *p* < 0.05 vs. the vehicle-treated UCMS group.

Serum corticosterone (CORT) levels were measured in order to clarify whether the feedback mechanism regulating endocrine secretion via the HPA axis was impaired by UCMS (Figure 4). Vehicle-treated UCMS mice had significantly higher serum CORT levels than non-stress control mice. However, UCMS-induced elevation in CORT levels was significantly suppressed by the daily treatment with imipramine (20 mg/kg/day) and the ethanol extract of *D. alatus* leaf (100–500 mg/kg/day) for 3 weeks (Figure 4) (for detailed statistical analysis, see Supplementary materials, Table S4).

### 2.4. Effect of and the Ethanol Extract of D. Alatus Leaf (LE) on Unpredictable Chronic Mild Stress (UCMS)-Induced Changes in Serum- and Glucocortoid-Inducible Kinase 1 (SGK1), Cyclic AMP-Responsive Element Binding (CREB) and Brain-Derived Neurotrophic Factor (BDNF) mRNA Expressions in Mice Frontal Cortex and Hippocampus 

**Figure 5:**
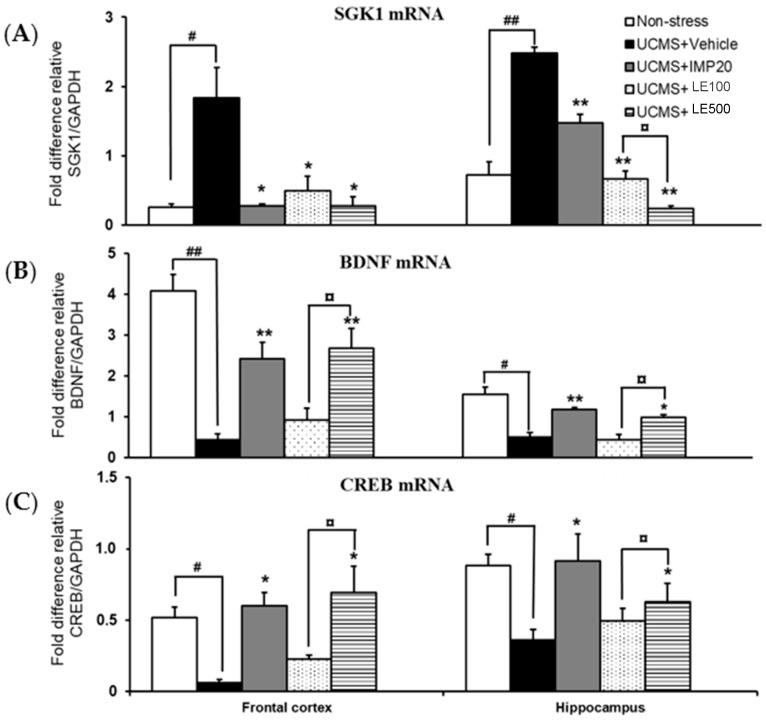
Effects of and the ethanol extract of *D. alatus* leaf (LE) on unpredictable chronic mild stress (UCMS)-induced changes in the frontal cortex and hippocampal expression of genes encoding serum- and glucocortoid-inducible kinase 1 (SGK1) (panel A), cyclic AMP-responsive element binding (CREB) (panel B), and brain-derived neurotrophic factor (BDNF) (panel C). Each column represents the mean ± S.E.M. (*n* = 3–5). ^*^ *p* < 0.05 and ^**^ *p* < 0.001 vs. the sham-operated group. ^#^ *p* < 0.05, ^##^ *p* < 0.001 vs. the UCMS group. ^¤^ *p* < 0.05 compared between doses of the ethanol extract of *D. alatus* leaf (LE).

As shown in Figure 5, quantitative real time PCR (QPCR) analysis of SGK1, CREB, and BDNF mRNAs expression revealed that the mRNA expression of SGK1 was significantly increased, whereas the mRNA expressions of CREB and BDNF were significantly increased in frontal cortex and hippocampus of UCMS mice. Following the treatments with the ethanol extract of *D. alatus* leaf (LE) and imipramine (IMP), the mRNA expression of SGK1 was markedly decreased in both of the brain regions, with a dose-dependent manner. Similar to the treatment with imipramine, the administration of and the ethanol extract of *D. alatus* leaf (LE) dose-dependently normalized the down-regulated expression of CREB and BNDF mRNAs in UCMS mice (for detailed statistical analysis, see Supplementary materials, Tables S5, S6, S7, and S8).

### 2.5. Monoamine Oxidase (MAO)-A and MAO-B Inhibitory Activities of the Ethanol Extract of D. alatus Leaf (LE)

The ethanol extract of *D. alatus* leaf (LE) was tested for the inhibitory activities on MAO-A and MAO-B, using the recombinant human MAO-A and MAO-B. The MAO-A/B mixed substrate, kynuramine, was used to measure the catalytic rates of the enzymes in the absence and the presence of various concentrations of test inhibitors. Kynuramine is oxidized by the MAOs to yield the fluorescent 4-hydroxyquinolone. Determination of the concentration of 4-hydroxyquinoline was conveniently performed by fluorescence spectrophotometry. The ethanol extract of *D. alatus* leaf (LE) showed inhibitory effects against MAO-A and MAO-B, with IC_50_ values of 197.7 ± 0.07 μM and 1158 ± 0.05 μM, respectively. The IC_50_ values were converted to the corresponding enzyme-inhibitor dissociation constants (*Ki* values) using the Cheng–Prusoff equation [21]; *Ki* = IC_50_/(1 + [S]/*Km*). For this purpose, *Km* values for the oxidation of kynuramine by MAO-A and MAO-B from previous studies, i.e., 16.1 μM for human MAO-A and 22.7 μM for human MAO-B, were used [22]. The *Ki* values allowed the calculation of the MAO-A/B selectivity ratios [Si = *Ki* (MAO-B/*Ki* (MAO-A)], and the results were shown in Table 1. The selectivity index for MAO-A and MAO-B isoforms indicates that the ethanol extract of *D. alatus* leaf (LE) was partially selective for MAO-A isoform, which is specific for antidepressant drugs.

**Figure 6:**
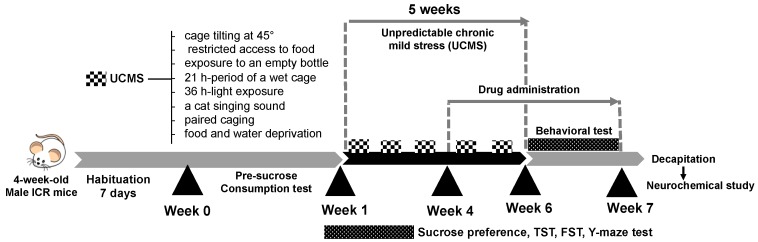
A schematic representation of the experimental protocols. The mice were divided into two main groups, i.e. the non-stress group and the unpredictable chronic mild stress (UCMS) group at week 1. The UCMS group was exposed to various unpredictable stressors from week 1 to week 6. The UCMS group was divided into 4 groups which daily received (i) vehicle, (ii) LE 100 mg/kg, (iii) LE 500 mg/kg, and (iv) IMP 20 mg/kg, from week 4 to week 7. Behavioral test started at week 6 to week 7. One day after the Y-maze task, mice were decapitated to collect the blood and brain for neurochemical analysis.

The experimental protocols are summarized as shown in Figure 6.

### 2.6. High Performance Liquid Chromatography (HPLC) Analysis of and the Ethanol Extract of D. alatus Leaf (LE) and Method of Validation

HPLC chromatograms from all standard solutions showed good separation of peaks, with resolution values more than 2 (Supplementary materials, Figure S1). The within-day and between-day percentage relative standard deviations (% RSD) for the five replicate injections of 1 µg/mL, 2 µg/mL, 3 µg/mL, 4 µg/mL, 5 µg/mL, and 6 µg/mL of standards were all less than 3% (Supplementary materials, Table S9). The calibration curve obtained from plotting the peak area at each concentration showed a good linearity (correlation coefficient r^2^ > 0.99, Supplementary materials, Table S9, Figure S1). Accuracy, as determined by the percentage recovery of spiked standards, was always between 90% and 110% (Supplementary materials, Table S1). The remaining validation data obtained for this HPLC method (limit of quantitation; LOQ and limit of detection; LOD) are presented in Table S9. From all validation parameters, it was found that this analytical method was reliable and could be used for the analysis of the six compounds.

HPLC was used to further determine the amount of the compounds in the ethanol extract of *D. alatus* leaf, and the retention times shown in Figure S1 were consistent with those of the standards. The major constituents of *D. alatus* leaf are flavonoids and phenolic acids. With this method, six compounds were identified, including luteolin-7-*O*-glucoside (12.51 ± 0.712 mg/g extract), kaempferol-3-glucoside (6.29 ± 0.084 mg/g extract), rutin (3.34 ± 0.056 mg/g extract), gallic acid (1.26 ± 0.009 mg/g extract), ferulic acid (1.04 ± 0.069 mg/g extract), and caffeic acid (0.84 ± 0.009 mg/g extract).

## 3. Discussion

This study aimed to investigate the antidepressant effect of in the ethanol extract of *D. alatus* leaf and its mechanism(s) of action, using UCMS mice as the animal model of depression. The present findings demonstrated that the ethanol extract of *D. alatus* leaf ameliorated UCMS-induced depressant behaviors by reversing hyperactivation of the HPA axis and neurogenesis in the hippocampus and frontal cortex of UCMS mice. The antidepressant effect of the ethanol extract of *D. alatus* leaf is similar to that of the classic antidepressant drug imipramine.

The UCMS is a well-known and effective model to mimic the pathogenesis of depression. In the UCMS procedure, animals are exposed to chronic and continuous low-grade stressors, similar to those associated with human depression. Several ethological symptoms and neurobiological abnormalities of the UCMS-induced animals are similar to those manifested in depressive patients [23]. In this study, UCMS mice exhibited the anhedonia behavior, as evidenced by the decrease in their sucrose preference. Anhedonia is a prominent symptom of depression which is defined as a decrease in responsiveness to rewards, as manifested by a reduction of the consumption of a sucrose solution. Treatment of the UCMS mice with the ethanol extract of *D. alatus* leaf during 2 weeks, by daily gavage, was able to reverse the decreased sucrose preference, thus confirming the antidepressant-like effect of the ethanol extract of *D. alatus* leaf. 

Immobility time is the characteristic behavior index in FST and TST, reflecting behavioral despair (hopeless behavior), as observed in depressive patients. A number of studies have demonstrated that mice exposed to repeated stress exhibited a longer immobility duration in the FST and TST [24,25]. Consistent with the previous findings, the present study also revealed the increased total time of immobility in both FST and TST in UCMS mice compared with that observed in non-stress control mice. The results showed that treatment with the ethanol extract of *D. alatus* leaf markedly reduced the immobility time of UCMS mice, similar to the established antidepressant effect of imipramine. Moreover, the locomotor activity in mice was also measured in order to rule out the possibility that the reduction in the immobility time caused by the extract might be due to an elevation in general locomotor activities. The result demonstrated that the ethanol extract of *D. alatus* leaf did not affect the locomotor activity, indicating that its antidepressant-like activity is specific. All the findings from behavioral experiments suggested that the ethanol extract of *D. alatus* leaf exhibited a significant antidepressant-like efficacy in the mouse model of depression, caused by UCMS exposure.

In order to explore the mechanism(s) of the antidepressant effect the ethanol extract of *D. alatus* leaf, the serum CORT levels were measured. It is well recognized that CORT, a biomarker of the HPA axis hyperactivation, plays an important role not only in therapeutic mechanisms of antidepressant drugs but also in the pathogenesis of endogenous depression [26,27]. As a chronic stressor can activate the HPA axis hyperactivity which leads to the release of CORT into the blood, treatment with some antidepressant drugs can attenuate the stress-induced elevation in serum CORT levels. Consistent with this hypothesis, the present study showed that mice exhibiting depression-like symptoms under the UCMS procedure had a significantly elevated level of serum CORT compared with the non-stress control mice. Interestingly, treatment with the ethanol extract of *D. alatus* leaf or imipramine was able to reverse this elevation. Moreover, the reduction of serum CORT by the ethanol extract of *D. alatus* leaf was dose-independent. Therefore, we concluded that the antidepressant-like effects of the ethanol extract of *D. alatus* leaf were due to the suppression of UCMS-induced HPA axis hyperactivation. 

SGK1, a serine/threonine kinase, is implicated in the neuronal function and cellular stress response. Previous reports have shown that GCs increased SGK1 expression in human neural stem cell, whereas an inhibitor of SGK1 hampered the glucocorticoid-induced reduction in neurogenesis, suggesting that SGK1 mediated the effects of GCs on glucocorticoid receptor function and neurogenesis and may be a key intermediary between stress and depression [11,28]. Anacker et al. have found that SGK mediated the cortisol-induced decrease in proliferation and neuronal differentiation of human progenitor cells by acting both downstream of glucocorticoid receptor activation, via SGK1-dependent inhibition of the Hedgehog pathway, and upstream glucocorticoid receptor activation, via SGK1-dependent glucocorticoid receptor phosphorylation and nuclear translocation [9]. Consistently, in the present study, exposure to UCMS significantly increased the mRNA expression of SGK1 in frontal cortex and hippocampus. Administration of the ethanol extract of *D. alatus* leaf and imipramine markedly suppressed an increase in the mRNA expression of SGK1 in both brain areas, in a dose-dependent manner. Interestingly, SGK1 reported a negative correlation with BDNF, which may evoke a potential mechanism of impaired neurogenesis in depression. All these evidences suggest that SGK1 is a crucial mediator of BDNF and neurogenesis inhibition by GCs. To further elucidate the pharmacological mechanism underlying the antidepressant effect of the ethanol extract of *D. alatus* leaf, its effects on BDNF signaling pathway were investigated. Previous studies suggested that exposure to chronic stress resulted in neural atrophy and reduced volume of hippocampus. Stress-induced inhibition of neurogenesis in the brain tissue is involved in the pathogenesis of depressive illness. Trophic factors, especially BDNF, was suggested to be associated with the development of depression, and thus responsible for the antidepressant activity in preclinical and clinical studies. UCMS caused an evident reduction of BDNF mRNA and protein expressions in the hippocampus and prefrontal cortex while chronic treatment with antidepressants obviously enhanced BDNF levels in those regions [29]. We also examined the CREB mRNA expression since CREB, a transcription factor, plays a critical role in neuronal plasticity and neurogenesis. Additionally, CREB upregulation may activate downstream targets, including BDNF, whose expression is dependent on CREB activation, and this process may be a key point of the therapeutic responses to antidepressants [30]. Consequently, we have found in this study that exposure to UCMS paradigm reduced the expression of CREB and BDNF mRNAs in the hippocampus and frontal cortex of mice. Administration of the ethanol extract of *D. alatus* leaf inhibited a decrease of CREB and BDNF mRNA expressions induced by UCMS paradigm, providing an evidence that elevation of BDNF and CREB expressions may contribute to the antidepressant property of the extract.

On the other hand, MAOs are the key enzymes involved in degradation of monoamine neurotransmitter. MAO-A catabolizes monoamine neurotransmitters including norepinephrine, serotonin, and dopamine. The abnormally elevated MAO-A activity could result in decreased levels of monoamine transmitters which, in turn, leads to depression [31]. Hence, brain MAO-A plays a major role in depressive disorders which could be considered as a target for the treatment of depression [32]. To further address the molecular mechanism in MAOs’ activities, the in vitro inhibitory effect of the ethanol extract of *D. alatus* leaf on MAO-A and MAO-B activities was also investigated in this study. The ethanol extract of *D. alatus* leaf was found to exhibit a partial selectivity for MAO-A. This finding suggested that the inhibitory effect on MAO-A may be one of the mechanisms of the ethanol extract of *D. alatus* leaf which are involved in the antidepressant activity.

Phytochemical analysis of the ethanol extract of *D. alatus* leaf by HPLC indicates the presence of flavonoids and phenolic acids as major constituents. These compounds were identified as luteolin-7-*O*-glucoside (12.51 ± 0.712 mg/g extract), kaempferol-3-glucoside (6.29 ± 0.084 mg/g extract), rutin (3.34 ± 0.056 mg/g extract), gallic acid (1.26 ± 0.009 mg/g extract), ferulic acid (1.04 ± 0.069 mg/g extract), and caffeic acid (0.84 ± 0.009 mg/g extract). Flavonoids have been shown to exert antidepressant property by various mechanisms of action. The major compound of the ethanol extract of *D. alatus* leaf is luteolin-7-*O*-glucoside. Luteolin exists as both aglycone and glycosides. Ishisaka et al. [33] reported the antidepressant-like effects of luteolin in behavioral tests. Moreover, luteolin also attenuated the expression of endoplasmic reticulum stress-related proteins in the hippocampus of corticosterone-treated depression-model mice. Oral administration of luteolin, at a dose of 50 mg/kg, resulted in antidepressant effects in FST and TST. Luteolin raised the levels of monoamine neurotransmitters in the synaptic cleft by directly and indirectly inhibiting serotonin uptake [34]. Kaemferol also exhibited the antidepressant activity by reducing the immobility time in FST and TST in stress-induced mice compared with unstressed mice [35]. Rutin, also known as quercetin-3-rutinoside, efficiently rescued the UCMS-induced behavioral deficits by reducing depression and anxiety and improving cognition. Rutin treatment also protected the UCMS-induced hippocampal neuronal loss, which might possibly explain its antidepressant activity [36]. Ferulic acid also exerts the antidepressant activity. Oral administration of ferulic acid, at a dose of 5 mg/kg for 7 days, significantly reduced immobility of ICR mice compared with vehicle-administered control group. Microarray and real-time PCR analyses revealed that ferulic acid upregulates the expression of several genes associated with cell survival and proliferation, energy metabolism, and dopamine synthesis in mice limbic system of brain [37]. These data support the results of antidepressant effect of *the* ethanol extract of *D. alatus* leaf in this study directly and indirectly.

## 4. Material and Methods

### 4.1. Preparation of the Leaf Extract of Dipterocarpus alatus

Leaves of *Dipterocarpus alatus* Roxb. Ex G. Don were collected from Khon Kaen University Campus, Thailand, in December 2016. The plant material was identified by Dr. Prathan Leucha of the Faculty of Pharmaceutical Sciences, Khon Kaen University, Thailand. The voucher specimen (PSKKU-PL024) was deposited at the herbarium of the Faculty of Pharmaceutical Sciences of Khon Kaen University, Thailand. Dried and powdered leaves (847.38 g) were macerated overnight in ethanol (3.5 L) at room temperature and filtered. The process was repeated 3 times, and the ethanol solutions were combined and evaporated, under reduced pressure at 50 °C, and freeze-dried to give the crude ethanol extract 60.35 g (7.12% yield) which was kept at −20 °C throughout the experiment.

### 4.2. Animals

Male ICR mice (20–30 g, 5 weeks old) were obtained from the National Laboratory Animal Center (Mahidol University, Nakhon Pathom, Thailand). Mice were housed on wood chip bedding in stainless steel cages with free access to food and water. Housing was thermostatically maintained at 22 ± 2 °C, with constant humidity (45% ± 2%) and a 12 h light-dark cycle (lights on: 06:00–18:00 h) in the Laboratory Animal Unit of the Faculty of Pharmaceutical Sciences, Khon Kaen University, Thailand. All behavioral experiments were performed from 07.30 to 17.00 h. The experimental protocols used in this study were in accordance with the Guiding Principles for the Care and Use of Animals (NIH Publications #8-23, revised in 2011) and were also approved by the Animal Ethics Committee for Use and Care of Khon Kaen Univeristy, Khon Kaen, Thailand (approval No. ACUC-KKU-15/2560).

### 4.3. Unpredictable Chronic Mild Stress (UCMS)

UCMS procedures were used to induce the depressive-like behaviors in mice as previously described by Mizuki et al. [38], with minor modification. Briefly, in week 0, the animals were first trained to consume a 2% sucrose solution for a 48-h period in their home cages with no food or water. After this training, the animals were deprived of water and food for 18 h, and then sucrose solution was given for 1 h per day for 5 consecutive days in order to group the mice and determine the baseline sucrose consumption. The animals were randomly divided into 5 groups: a non-stressed control group and 4 other groups subjected to UCMS for 5 weeks. The UCMS procedures consisted of a variety of unpredictable mild stressors, including two periods of cage tilting at 45° (12 h), two periods of restricted access to food (5 micropellets, 1 h), two periods of exposure to an empty bottle (3 h), one 21 h-period of a wet cage (200 mL of water in 100 g of sawdust bedding), two periods of light exposure (36 h), two periods of intermittent sound (a cat singing sound) (3 and 5 h), two periods of paired caging (2 h), and food and water deprivation for 18 h before the measurement of 2% sucrose solution consumption. These stressors were randomly scheduled over a one-week period and repeated throughout the 5-week experiment. The non-stressed control group was housed under normal conditions.

### 4.4. Drug Administration

Vehicle (0.5% sodium carboxymethyl cellulose, SCMC) and drugs were daily administrated at week 4 to week 7 (Figure 6) at 8.00 am. On a behavioral testing day, all treatments were conducted 1 h before testing. The mice were divided into 5 groups (*n* = 12). The 1st group was a non-stress group (0.5% SCMC, 1 mL/kg, p.o.). The 2nd group was a stressed or UCMS group (0.5% SCMC, 10 mL/kg, p.o.). The 3rd group was the UCMS which received imipramine hydrochloride (IMP) 20 mg/kg, i.p. The 4th and 5th groups were UCMS groups which received LE 100 and LE 500 mg/kg, p.o. The ethanol extract of *D. alatus* leaf extract (LE, 1 g) was freshly suspended in 0.5% SCMC (10 mL) and used as a stock solution. The reference standard drug, imipramine hydrochloride (IMP) (Sigma-Aldrich, St. Louis, MO, USA) was dissolved in 0.9% saline. One day after finishing behavioral test, the mice were decapitated. All tissues were collected immediately and kept at −80 °C throughout the experiment.

### 4.5. Sucrose Preference Test

The sucrose preference test was used to assess the anhedonic-like behavior. The test was conducted once a week after the end of the UCMS procedure. Before the test, mice were individually put in a cage and deprived of food and water for 18 h. In the test, mice received 2% (*w/v*) sucrose solution for 1 h. The amounts of 2% (*w/v*) sucrose solution were recorded [38]. Food and water were available *ad libitum* after the sucrose preference test. 

### 4.6. Tail Suspension Test (TST)

Each mouse was suspended upside down in the tail suspension test box, which leads to characteristic behavior immobility [25]. After the treatment, mice were suspended by adhesive tape, placed approximately 1 cm from the tip of the tail on the edge of TST apparatus which is 30 cm hung above the floor. Immobility duration was recorded for the last 4 min, during a 6-min observation period. The immobility behavior of mice considered by being hung passively and completely motionless [39].

### 4.7. Forced Swimming Test (FST)

Forced swimming test (FST) is one of the most commonly used animal models for assessing antidepressant-like behavior. FST involves the scoring of active (swimming and climbing) or passive (immobility) behavior when mice were forced to swim in a cylinder from which there is no escape [40]. Mice were placed individually in a transparent glass cylinder (12 cm in diameter, 25 cm in height) which was filled with water to the height of 10 cm at 25 °C. For the pre-test session, mice were forced to swim for 15 min in a pre-swim session, after which they were allowed to dry and then return to their cages. After 24 h of resting, the mice were forced to swim for 5 min in a test session. In the test session, each mouse was administered drug 1 h before the test and was placed in the cylinder. The immobility time was recorded during 5 min.

### 4.8. Locomotor Test

In order to exclude false positive results from drug-induced hyperlocomotion in hopeless behavioral tests, locomotor activity was determined by evaluation of mice movement. The Y-maze task was used to determine the locomotor activity. The Y-maze consisted of three equal arms 40 cm long, 18 cm high, 3 cm wide at the bottom, and 12 cm wide at the top, which were positioned at equal angles as Y-shape. One hour after drug administration, the mice were individually placed on one arm, and the total arm entries were recorded manually over 8 min-period for measuring the locomotion activity [41].

### 4.9. Serum Corticosterone Assay

After completing the behavioral analysis, blood was collected via cardiac puncture under anesthesia with pentobarbital sodium as anesthetic drug (Nembutal®: 60 mg/kg i.p.; Ceva Sante Animale, Libourne, France). The blood was left overnight at room temperature and centrifuged at 3000 rpm at 21 °C for 20 min. Assay Max Corticosterone ELISA kit (Assaypro LLC, St. Charles, MO, USA) was used to determine the serum corticosterone level according to the previously described procedure [41]. The serum sample (25 µL) was added in 96-well microplates of ELISA kit and immediately added 25 µL of biotinylated protein to each well. The 96-well microplates were gently mixed and incubated for 2 h at room temperature and then washed with 200 µL of washing solution for five times, and added 50 µL of streptavidin-peroxidase conjugate into each well before incubation for 30 min. Each well was washed with 200 µL of washing solution for five times again and then added 50 μL of chromogen substrate and incubated for 20 min. The reaction was stopped by adding 50 µL of stop solution. The absorbance was immediately determined at 450 nm. 

### 4.10. Quantitative Real Time PCR (QPCR)

Mouse serum/glucocorticoid-regulated kinase 1 (SGK1), cyclic AMP response element binding protein (CREB), brain-derived neurotrophic factor (BDNF), mRNAs expression in hippocampus and frontal cortex were quantified by real time PCR. Total RNA was extracted from the tissues with TRIzol® (Thermo Fisher Scientific Inc., San Jose, CA, USA), according to the manufacturer’s instructions. First-strand cDNA was synthesized with oligo (dT) primers and SuperScript III reverse transcriptase (Thermo Fischer Scientific Inc., San Jose, CA, USA). QPCR was conducted using SsoAdvanced™ Universal SYBR® Green Supermix (Biorad, Hercules, CA, USA). The following primers were synthesized by Macrogen (Seoul, South Korea): amplification was carried out using gene-specific PCR primer sets as shown in Table 2. Glyceraldehyde-3-Phosphate Dehydrogenase (GAPDH) mRNA was used as a control to which the results were normalized. Fold difference relative expressions were calculated.

### 4.11. Human Monoamine Oxidase (MAO)-A and MAO-B Inhibitory Activity Assay

The recombinant human MAO-A and MAO-B (Sigma-Aldrich, St. Louis, MO, USA) inhibitory activity assays of the *D. alatus* leaf extract were performed according to the previously described procedure [41]. Briefly, 100 mg of the crude extract was dissolved in 400 µL DMSO to prepare 250 mg/mL as a stock solution. Serial dilution of test samples (20 µL) for each concentration was used to test the activity. Concentration of MAOs was 0.0075 mg/mL for MAO-A inhibitor test, 9 µL of kynuramine (Sigma-Aldrich, St. Louis, MO, USA) was mixed with 469.5 µL of potassium phosphate buffer (100 mM, pH 7.4, made isotonic with KCl, 20.2 mM). For MAO-B inhibitor test, 6 µL of kynuramine was mixed with 472.5 µL of potassium phosphate buffer (pH 7.4). Then 1.5 µL of the enzymes were added before incubation at 37 °C for 20 min. The reaction was stopped by adding 400 µL of 2N NaOH and 1000 µL of water. Measurement of 4-hydroxyquinoline (Sigma-Aldrich, St. Louis, MO, USA) was carried out by fluorescence spectrophotometry with the excitation wavelength at 310 nm and the emission wavelength at 400 nm. Sigmoidal dose-response curves were constructed by plotting the initial rates of MAO-catalyzed kynuramine oxidation versus the logarithm of the inhibitor concentrations and expressed as IC_50_ values which were determined in duplicate and expressed as mean ± standard deviation (SD). Data were analyzed by Prism 5 software package (GraphPad Software, version 5, San Diego, CA, USA). The IC_50_ values were converted to the corresponding *Ki* values according to the equation *Ki* = IC_50_/(1 + [S]/*Km*). Clorgyline (Sigma-Aldrich, St. Louis, MO, USA) and deprenyl (Sigma-Aldrich, St. Louis, MO, USA) were used as control for selective MAO-A and MAO-B inhibitors, respectively.

### 4.12. High-Performance Liquid Chromatography (HPLC) Analysis and Validation of Analytical Method

The leaf extract of *D. alatus* was analyzed by a reversed-phase HPLC system using a Hypersil ODS column (Agilent Technologies Inc., Santa Clara, CA, USA, 4 × 250 mm, 5 µm). Methanol (Solvent A) and 0.2% formic acid in aqueous solution (solvent B) were used as a mobile phase, with gradient elution (30–60% solvent A, cycle time 65 min.) with the flow rate of 1.2 mL/min. Standards were detected at 254, 275, and 370 nm, as previously described [42,43]. The amount of gallic acid, caffeic acid, ferulic acid, luteolin-7-*O*-glucoside, rutin, and kaempferol-3-glucoside present in the extract was then determined by comparison with standard curves (1–6 µg/mL). The analytical method was validated for specificity by the absence of undesired peaks in HPLC chromatograms and accuracy by the percentage recovery of all standards in 5 replicates. Precision (%RSD) was validated for within-day and between-day determinations (*n* = 5. Linearity was validated by using linear regression analysis to calculate the coefficient of determination (r2) of standard curve (1–6 µg/mL, *n* = 5). Limit of detection (LOD) and limit of quantitation (LOQ) were also determined, and signal to noise ratios were calculated (*n* = 5).

### 4.13. Statistical Analysis

The data obtained from in vitro studies were expressed as the mean ± SD. Behavioral and neurochemical data were expressed as the mean ± S.E.M. and were examined by paired Student’s t-test for non-stressed versus UCMS group or one-way analysis of variance (ANOVA), followed by Tukey’s post hoc test for multiple comparisons. Differences of *p* < 0.05 were considered to be statistically significant. The analysis was conducted using SigmaStat® ver. 3.5 (SYSTAT Software Inc., San Jose, CA, USA).

## 5. Conclusions

In the present study, we first demonstrated the antidepressant-like effect of the ethanol extract of *D. alatus* leaf in UCMS mice model of depression. Treatment with the ethanol extract of *D. alatus* leaf ameliorated the anhedonia and hopeless behaviors in UCMS mice. Notably, our results strongly suggested that the reduced serum CORT levels, as well as the restored SGK1, CREB, and BDNF expressions in the frontal cortex and hippocampus, may be involved in the therapeutic action of the ethanol extract of *D. alatus* leaf in depression. In addition, the ethanol extract of *D. alatus* leaf also exerts the selective inhibitory effect on MAO-A. These findings are relevant for understanding the antidepressant mechanism of *D. alatus* leaf extract. This study revealed that *D. alatus* leaves could be a new resource that can have potential application as nutraceuticals (or functional additives) with depression-regulating properties. However, further studies are necessary to clarify the precise mechanism for promoting neurogenesis in dentate gyrus of hippocampal and other signaling pathways in HPA axis function.

## Figures and Tables

**Table 1:** Inhibitory effects of and the ethanol extract of *D. alatus* leaf, clorgyline and deprenyl on monoamine oxidase (MAO)-A and MAO-B activities.

Extract/Compounds	IC_50_ (μM)		*Ki* (μM)		Si	
	MAO-A	MAO-B	MAO-A	MAO-B	MAO-A	MAO-B
*D. alatus* leaf extract (LE)	197.70 ± 0.07 µg/mL	1158.00 ± 0.05 µg/mL	52.09	498.80	0.10	0.09.58
Clorgyline *	0.14 ± 0.04	1.40 ± 0.24	0.037	0.603	0.061	16.276
Deprenyl **	950.20 ± 0.14	0.21 ± 0.08	250.380	0.087	2869.095	0.000348

All data are expressed as mean ±SD. * Clorgyline is a reference for selective monoamine oxidase (MAO)-A inhibitor, ** Deprenyl is a reference for selective MAO-B inhibitor. *Ki* is the corresponding enzyme-inhibitor dissociation constant and Si is the selectivity index between MAO-A and MAO-B.

**Table 2:** Summary of primer sequences used for quantitative real-time PCR (QPCR).

Gene	Primer Sequence	Fragment Size
SGK1	Forward: 5′-GGG TGC CAA GGA TGA CTT TA-3′ Reverse: 5′-CTC GGT AAA CTC GGG ATC GA-3′	154 bp
CREB	Forward: 5′-TAC CCA GGG AGG AGC AAT AC-3′ Reverse: 5′-GAG GCA GCT TGA ACA ACA AC-3′	183 bp
BDNF	Forward: 5′-GAC AAG GCA ACT TGG CCT AC-3′ Reverse: 5′-CCT GTC ACA CAC GCT CAG CTC-3′	334 bp
GAPDH	Forward: 5’-ACC ACA GTC CAT GCC ATC AC-3’ Reverse: 5’-TCC ACC ACC CTG TTG CTG TA-3’	452 bp

## Supplementary Materials

**Table S1**. One-way analysis of variance (ANOVA) test of sucrose preference test. **Table S2**. One-way analysis of variance (ANOVA) test of FST. **Table S3**. One-way analysis of variance (ANOVA) test of TST. **Table S4**. One-way analysis of variance (ANOVA) test of serum corticosterone (CORT) levels. **Table S5**. One-way analysis of variance (ANOVA) test of SGK1 mRNA expression (Frontal cortex). **Table S6**. One-way analysis of variance (ANOVA) test of SGK1 mRNA expression (Hippocampus). **Table S7**. One-way analysis of variance (ANOVA) test of BDNF mRNA expression (Frontal cortex). **Table S8**. One-way analysis of variance (ANOVA) test of BDNF mRNA expression (Hippocampus). **Figure S1**. HPLC chromatograms of six standards’ solution (A) and D. alatus leaf extract (B) (1 = Gallic acid, 2 = Caffeic acid, 3 = Ferulic acid, 4 = Luteolin-7-O-glucoside, 5 = Rutin, 6 = Kaempferol-3-glucoside). **Table S9**. Validation results of the analytical method for determination of flavonoids and phenolic acids.
