# Supplementary material for: Effects of the Ethanol Extract of Dipterocarpus alatus Leaf on the Unpredictable Chronic Mild Stress-Induced Depression in ICR Mice and Its Possible Mechanism of Action
Source: Molecules. 2019 Sep 18;24(18):3396. doi: 10.3390/molecules24183396 (PMC6767234; doi:10.3390/molecules24183396)
Supplement: Supplementary file 1 [file molecules-24-03396-s001.pdf]

Supplementary Materials

Effects of the Ethanol Extract of *Dipterocarpus alatus* Leaf on the Unpredictable Chronic Mild Stress-Induced Depression in ICR Mice and Its Possible Mechanism of Action

Supawadee Daodee[1], Orawan Monthakantirat[1], Kanlaya Ruengwinitwong[1], Kankrittanon Gatenakorn[1], Juthamart Maneenet[1], Charinya Khamphukdee[1], Nazim Sekeroglu[2], Yaowared Chulikhit[1*] and Anake Kijjoa[3*]

[1] Division of Pharmaceutical Chemistry, Faculty of Pharmaceutical Sciences, Khon Kaen University, Khon Kaen 40002, Thailand. E-mail: csupawad@kku.ac.th (S.D.); oramon@kku.ac.th (O.M.); k.ruengt@gmail.com (K.R.); kankrittanon@gmail.com (K.G.); juthamart_pp@hotmail.com (J.M.); charkh@kku.ac.th (C.K.); yaosum@kku.ac.th (Y.Ch.).

[2] Department of Horticulture, Faculty of Agriculure, Killis 7 Aralik University, Killis 79000, Turkey. E-mail: nsekeroglu@gmail.com.

[3] *ICBAS*-Instituto de Ciências Biomédicas Abel Salazar and CIIMAR, Rua de Jorge Viterbo Ferreira, 228, 4050-313 Porto, Portugal. E-mail: ankijjoa@icbas.up.pt.

*To whom correspondence should be addressed, E-mail: yaosum@kku.ac.th (Y.Ch.) and ankijjoa@icbas.up.pt (A.K.), Tel.: +351-220428331 (A.K.)

Table S1. One-way analysis of variance (ANOVA) test of sucrose preference test.

Group comparison	ANOVA. followed by Tukey's post hoc test	
	P	F ($DF_{between\ group}$, $DF_{residual}$)
All group	<0.001	F (4,40) =13.218
Non-stress group VS. vehicle-treated UCMS group	<0.001	
Vehicle-treated UCMS group VS. UCMS+IMP	0.0019	
Vehicle-treated UCMS group VS. UCMS+LE100	0.001	
Vehicle-treated UCMS group VS. UCMS+LE500	<0.001	

Table S2. One-way analysis of variance (ANOVA) test of FST.

Group comparison	ANOVA followed by Tukey's post hoc test	
	P	F ($DF_{between\ group}$, $DF_{residual}$)
All group	<0.001	F (4,44) =27.974
Non-stress group VS. vehicle-treated UCMS group	<0.001	
Vehicle-treated UCMS group VS. UCMS+IMP	<0.001	
Vehicle-treated UCMS group VS. UCMS+LE100	<0.001	
Vehicle-treated UCMS group VS. UCMS+LE500	<0.001	
UCMS+LE 100 VS. UCMS+LE 500	0.0012	

Table S3. One-way analysis of variance (ANOVA) test of TST.

Group comparison	ANOVA followed by Tukey's post hoc test	
	P	F ($DF_{between\ group}$, $DF_{residual}$)
All group	<0.001	F (4,45) =15.423
Non-stress group VS. vehicle-treated UCMS group	<0.001	
Vehicle-treated UCMS group VS. UCMS+IMP	<0.001	
Vehicle-treated UCMS group VS. UCMS+LE100	<0.001	
Vehicle-treated UCMS group VS. UCMS+LE500	<0.001	

Table S4. One-way analysis of variance (ANOVA) test of serum corticosterone (CORT) levels.

Group comparison	ANOVA followed by Tukey's post hoc test	
	P	F ($DF_{between\ group}$, $DF_{residual}$)
All group	<0.001	F (4,16) =8.092
Non-stress group VS. vehicle-treated UCMS group	<0.001	
Vehicle-treated UCMS group VS. UCMS+IMP	0.036	
Vehicle-treated UCMS group VS. UCMS+LE100	0.564	
Vehicle-treated UCMS group VS. UCMS+LE500	0.001	

Table S5. One-way analysis of variance (ANOVA) test of SGK1 mRNA expression (Frontal cortex).

Group comparison	ANOVA followed by Tukey's post hoc test	
	P	F (DF$_{\text{between group}}$, DF$_{\text{residual}}$)
All group	0.022	F (2,6) =7.22
Non-stress group VS. vehicle-treated UCMS group	0.005	
Vehicle-treated UCMS group VS. UCMS+IMP	0.017	
Vehicle-treated UCMS group VS. UCMS+LE100	0.017	
Vehicle-treated UCMS group VS. UCMS+LE500	0.017	

Table S6. One-way analysis of variance (ANOVA) test of SGK1 mRNA expression (Hippocampus).

Group comparison	ANOVA followed by Tukey's post hoc test	
	P	F (DF$_{\text{between group}}$, DF$_{\text{residual}}$)
All group	<0.001	F (4,16) = 67.663
Non-stress group VS. vehicle-treated UCMS group	<0.001	
Vehicle-treated UCMS group VS. UCMS+IMP	<0.001	
Vehicle-treated UCMS group VS. UCMS+LE100	<0.001	
Vehicle-treated UCMS group VS. UCMS+LE500	<0.001	
UCMS+LE 100 VS. UCMS+LE 500	0.016	

Table S7. One-way analysis of variance (ANOVA) test of BDNF mRNA expression (Frontal cortex).

Group comparison	ANOVA followed by Tukey's post hoc test	
	P	F (DF$_{\text{between group}}$, DF$_{\text{residual}}$)
All group	<0.001	F (4,10) =13.705
Non-stress group VS. vehicle-treated UCMS group	<0.001	
Vehicle-treated UCMS group VS. UCMS+IMP	<0.001	
Vehicle-treated UCMS group VS. UCMS+LE500	<0.001	
UCMS+LE 100 VS. UCMS+LE 500	0.016	

Table S8. One-way analysis of variance (ANOVA) test of BDNF mRNA expression (Hippocampus).

Group comparison	ANOVA followed by Tukey's post hoc test	
	P	F (DF$_{\text{between group}}$, DF$_{\text{residual}}$)
All group	0.006	F(4,12)=11.526
non-stress group VS. vehicle-treated UCMS group	0.006	
vehicle-treated UCMS group VS. UCMS+IMP	<0.001	
vehicle-treated UCMS group VS. UCMS+LE500	0.049	
UCMS+LE 100 VS. UCMS+LE 500	0.016	

Figure S1. HPLC chromatograms of six standards' solution (A) and *D. alatus* leaf extract (B) (1 = Gallic acid, 2 = Caffeic acid, 3 = Ferulic acid, 4 = Luteolin-7-*O*-glucoside, 5 = Rutin, 6 = Kaempferol-3-glucoside).

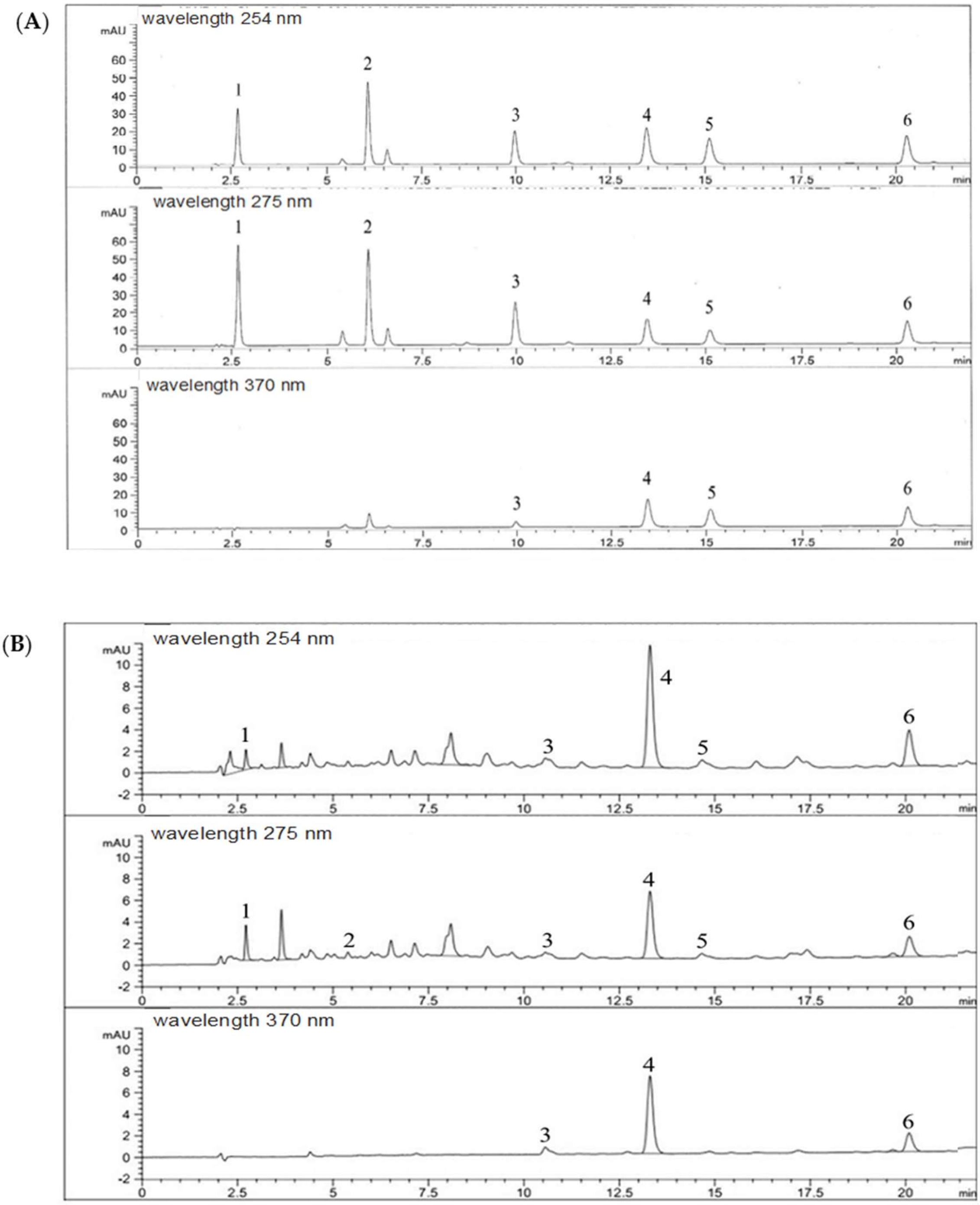

Table S9. Validation results of the analytical method for determination of flavonoids and phenolic acids.

**Parameter**		Compounds					
		Gallic acid	Caffeic acid	Ferulic acid	Luteolin-7-*O*-glucoside	Rutin	Kaempferol-3-glucoside
**Linearity**	Range (μg/mL)	1-6	1-6	1-6	1-6	1-6	1-6
	Coefficient Determination (R^2)	0.9987±0.0012	0.9949±0.0031	0.9984±0.0013	0.9988±0.0014	0.9951±0.0023	0.9938±0.0051
LOD (μg/mL)		0.5 (S/N~ 4.04±0.71)	0.5 (S/N~ 3.88±0.90)	0.5 (S/N~ 3.21±0.71)	0.5 (S/N~ 4.28±0.43)	0.5 (S/N~ 4.03±0.35)	0.5 (S/N~ 3.81±0.28)
LOQ (μg/mL)		1 (S/N~ 11.15±1.32)	1 (S/N~ 12.26±1.37)	1 (S/N~ 11.05±1.74)	1 (S/N~ 12.85±1.30)	1 (S/N~ 12.09±1.04)	1 (S/N~ 11.44±0.83)
Precision (%RSD)	Within day	0.15-0.38	1.33-2.99	0.42-1.39	0.60-1.99	0.01-1.40	0.62-3.76
	Between day	2.84-5.77	1.43-5.34	1.23-3.46	0.75-5.76	1.79-6.73	0.30-4.89
Accuracy % Recovery	Conc. 1 μg/mL	100.29±1.66	100.49±3.95	99.73±0.81	100.23±6.29	109.54±2.78	104.83±3.58
	Conc. 3 μg/mL	100.60±3.54	99.05±3.98	98.78±3.53	100.43±4.89	96.34±3.21	94.15±4.930
	Conc. 6 μg/mL	99.92±0.93	97.67±1.63	100.73±2.03	99.32±1.88	102.05±1.79	104.73±2.94
